# Effects of monobutyrin supplementation on egg production, biochemical indexes, and gut microbiota of broiler breeders

**DOI:** 10.1016/j.psj.2020.11.074

**Published:** 2020-12-10

**Authors:** Xin Feng, Fangang Kong, Liwei Zheng, Qien Qi, Lina Long, Li Gong, Weilong Huang, Huihua Zhang

**Affiliations:** ∗ School of Life Science and Engineering, Foshan University, Foshan, China 528000; † Kaiping Lvhuang Agriculture and Animal Husbandry Development Co., Ltd., Jiangmen, China 529311

**Keywords:** monobutyrin, Qingyuan partridge chicken, production, microbiota

## Abstract

The objective of the present study was to determine the effect of monobutyrin supplementation on egg production, biochemical indexes, and gut microbiota of broiler breeders at the late stage of production. A total of 180 healthy Qingyuan partridge broilers were randomly assigned to 2 groups: 1) corn–soybean meal–based diet and 2) basal diet supplemented with 250 mg monobutyrin/kg. Each treatment group had 6 replicates/cages with 15 birds within each replicate. The experiment started at week 33 and lasted for 8 wk. Egg production rate, feed conversion rate, shell breaking strength, and shell thickness were not different between control and treatment groups. Supplementation of monobutyrin increased egg weight and tended to decrease egg breaking rate of Qingyuan partridge chickens. Supplementation of monobutyrin did not affect any of the biochemical indexes except total protein concentration. The 4 antioxidant parameters measured were not affected either. Alpha diversity indexes (Shannon, Simpson, Chao1, Ace, and Good’s Coverage) and composition of cecal microbiota were not affected by monobutyrin supplementation. Overall, supplementation of monobutyrin at 250 mg/kg level improved egg quality, but its effect on cecal microbiota composition was limited.

## Introduction

Maintaining a healthy intestinal development is critical to ensure improved growth performance and health of the animals. The poultry production sector is prompted to find alternatives as the preventive use of antibiotics has become more and more restricted. Butyric acid has been reported to have antibacterial effects and promote growth in animals. It belongs to the short-chain fatty acid group and can diffuse through bacterial cell membranes and dissociate inside the bacterial cell, leading to a drop of intracellular pH of the bacterial cell and eventually death (Hanna, 2019).

Butyrate was recommended by poultry nutritionists to support intestinal health and enhance growth performance of broiler chickens (Moquet, 2018). Bedford et al. (2017) studied effects of monobutyrin on broiler chickens and reported that monobutyrin did not affect ADG and feed efficiency. However, birds in the 2,000-ppm monobutyrin group had significantly lower abdominal fat deposition than birds in the control group. Antongiovanni et al. (2007) observed that slaughtering weight and feed efficiency of broiler chickens were increased with butyrate glyceride supplementation. Yin et al. (2016) reported that mixture of butyrate glycerides (mainly monobutyrin and tributyrin) improved the performance of broiler chickens, especially on lipid catabolism.

The avian gastrointestinal tract harbors a complex microbiota that influences nutrition and health of the host. A balanced gut microflora is necessary to provide additional nutrients and protection against pathogens (Gabriel et al., 2006). Dysbiosis of broiler chickens arise because of the withdrawal of antibiotic growth promoters, diet changes, and environmentally induced stress in modern broiler production. Butyric acid derivatives have been incorporated into diets of broilers to replace antibiotics and reported to decrease *Salmonella* Enteritidis infection and improve growth performance under stress (Zhang et al., 2011). Leeson et al. (2005) reported that butyrate glycerides can maintain the broiler performance during coccidiosis challenge. Bedford and Gong (2018) suggested using butyrate as an additive to combat gastrointestinal tract disorders to improve gut health and performance of chickens. Feeding tributyrin can also increase expression of several tight junction proteins such as E-cadherin and zonula occludens-1 (Moquet, 2018).

The alpha monoglycerides of these short-chain fatty acids are reported to have stronger antibacterial effects (Namkung et al., 2011) and supplementation in the diet might benefit chicken gut health and growth performance. There have been many studies conducted on how butyric acid and its other forms affect growth performance in broilers. However, limited studies have been conducted on the effects of butyrate on egg quality, profiles of blood indexes, and gut microflora of broiler breeders. The objective of the present study was to investigate how butyrate glyceride (monobutyrin) affect egg quality, blood parameters, and cecal microflora of broiler breeders after the peak production period.

## Materials and methods

### Ethics Statement

This experimental protocol was approved by the Ethical Committee and conducted under the supervision of the Institutional Animal Care and Use Committee of Foshan University (Foshan, China).

### Experimental Design and Diet

A total of 180 healthy Qingyuan partridge broilers (33 wk) were randomly assigned to 2 groups: 1) corn–soybean meal–based diet and 2) basal diet supplemented with 250 mg monobutyrin/kg. Each treatment group had 6 replicates/cages with 15 birds in each replicate. The experiment started at week 33 and lasted for 8 wk. The basal diet was formulated as per the nutrient requirements for laying hens (2012), and the feed ingredients and dietary nutrient compositions are presented in Table 1. During the study, the birds had free access to feed and drinking water. The room was cleaned and disinfected daily, and the house was controlled at constant temperature and maintained on a 16-hour light regime.

**Table 1:** Feed ingredients and nutrient composition of the basal diet.

Feed ingredients	%	Nutrient composition	%
Corn	60.80	ME (kcal/kg)	4,041.6
Soybean meal	26.00	CP	17.00
Limestone	7.74	Calcium	3.25
Soybean oil	2.62	Phosphorus	0.50
Calcium bicarbonate	1.40	Salt	0.03
Lysine	0.18	Lysine	0.998
DL-Methionine	0.18	DL-Methionine	0.435
Threonine	0.08		
1% Premix	1.00		
Total	100		

1% premix includes the following: vitamin A, 12,000 IU; vitamin D_3,_ 5,000 IU; vitamin B_2,_ 25 mg; vitamin K, 2 mg; vitamin E, 30 mg; vitamin B, 3 mg; vitamin B_12,_ 1 mg; niacin 3 g; pantothenic acid 800 mg; folic acid, 500 mg; biotin, 0.2 mg; choline, 1,500 mg; Fe, 10 mg; Cu, 8 mg; Mn, 10 mg; I, 42 mg; Se, 30 mg.

### Production Performance and Egg Quality

Feed intake and number of eggs were recorded daily from week 33 to 40, and feed conversion rate was calculated. The number of broken eggs was recorded, and egg breaking rate was calculated. In the last week of the study, 6 eggs from each replicate (36 eggs per treatment) were randomly selected, and egg weight (Egg Analyzer; Orka Food Technology Ltd., Israel), shell breaking strength (Egg Force Reader; Orka Food Technology Ltd., Israel), and shell thickness (Eggshell Thickness Gauge; Orka Food Technology Ltd., Israel) were determined. Egg shape index (%) was calculated as the egg width-to-length ratio. All analyses were conducted by 1 trained person blind to the treatments.

### Blood Sample Collection and Analysis

At the end of the study, 1 bird was randomly selected from each replicate. The blood sample was collected from the wing vein and analyzed for total protein, total cholesterol, albumin, triglyceride, alkaline phosphate, and calcium. Antioxidant parameters including malondialdehyde, total antioxidant capacity, superoxide dismutase, and glutathione peroxidase were determined as per the instructions provided with the kits (Nanjing Jiancheng Bioengineering Inc., China). The selected chickens were then sacrificed by cervical dislocation and exsanguinated. The digesta from right and left cecum (pooled within broiler) were aseptically collected from each individual broiler and immediately placed into capped vials. The samples were stored at −80°C until further analysis.

### Cecal Digesta DNA Extraction and High-Throughput Sequencing Analysis

Total genome DNA from cecal digesta was extracted using the cetyltrimethylammonium bromide method (Trojanek et al., 2017). Extracted DNA was monitored on 1% agarose gels before being diluted to 1 ng/μL to prepare amplicons for high-throughput sequencing. Conventional PCR was used to amplify the V4 regions of the 16S rRNA genes using primers 515F (5′- GTGYCAGCMGCCGCGGTAA-3′) and 806R (5′-GGACTACNNGGGTATCTAAT-3′). The PCR reaction mix consisted of 15 μL of Phusion High-Fidelity PCR Master Mix (New England Biolabs), 0.2 μmol of forward and reverse primers, and about 10 ng template DNA. Reaction condition consisted of initial denaturation at 98°C for 1 min, followed by 30 cycles of denaturation at 98°C for 10 s, annealing at 50°C for 30 s, elongation at 72°C for 30 s, and a final extension at 72°C for 5 min. The PCR products were mixed with the same volume of 1X loading buffer (contained SYB green), then examined on 2% agarose gene. Only samples with bright strip between 400 and 450 bp were chosen for further analysis. Sequencing libraries were generated using TruSeq DNA PCR-Free Sample Preparation kit (Illumina) following the manufacturer's recommendations, and index codes were added. The library quality was assessed on a Qubit @ 2.0 Fluorometer (Thermo Fisher Scientific) and Agilent Bioanalyzer 2100 system (Agilent Technologies, Inc.). The bar-coded amplicons were sequenced on an Illumina NovaSeq system and 250-bp paired-end reads were generated.

Paired-end reads were merged using Fast Length Adjustment of SHort reads software (V1.2.7) (Magoc and Salzberg, 2011), and quality filtering on the raw sequences were conducted on a quality control pipeline using the Quantitative Insight into Microbial Ecology tool kit to obtain the high-quality clean reads (Caporaso et al., 2010; Bokulich et al., 2013). Chimera sequences were removed by comparing with the Silva database using UCHIME algorithm (Edgar et al., 2011; Haas et al., 2011). The effective tags were retained for analysis. The obtained high-quality reads were assigned to the same operational taxonomic units (**OTU**) at ≥97% similarity using the Quantitative Insight into Microbial Ecology UCLUST algorithm (Edgar, 2013). Taxonomic analysis was performed at the phylum and genus levels. Operational taxonomic unit abundance information was normalized, and subsequent diversity analysis was performed using the normalized data. Alpha diversity analysis (Shannon, Simpson, Chao1, Ace, and Good’s Coverage) was conducted to study the complexity of species diversity using Quantitative Insight into Microbial Ecology (V1.9.1). Principal coordinate analysis was performed to get principal coordinates with Bray–Curtis distance algorithm, and the data were displayed by WGCNA and ggplot2 packages in R software (V4.0.0; R Core Team, 2013).

### Statistical Analysis

All data were analyzed using the PROC GLIMMIX procedure of SAS (SAS Institute, Inc., Cary, NC) with treatment as fixed effect in the model. The significance was declared at *P* < 0.05 and trends at *P* < 0.1.

## Results and discussion

### Production and Egg Quality

For broiler breeders, egg production and egg quality are of great economic concern. Eggshell strength is one of the important egg qualities, and maintaining a high egg shell breaking strength is necessary for lower economic losses for producers. In present study, supplementation of monobutyrin did not affect egg production rate, feed-to-egg ratio from week 33 to week 40 (*P* > 0.05; Table 2). However, egg weight was significantly increased in the treatment group compared with the control group (48.73 vs. 50.74; *P* = 0.043). The egg breaking rate in the treatment group tended to be lower than that inthe control group (*P* = 0.07). Egg shape index, shell breaking strength, and shell thickness were not different between 2 groups (*P* > 0.05).

**Table 2:** Effects of monobutyrin supplementation on production and egg quality of broiler breeders.

Item	CG	BY	SEM	*P* value
Egg production rate%	65.29	68.51	1.449	0.15
Feed:egg, g/g	2.89	2.79	0.077	0.35
Egg weight, g	48.73	50.74	0.614	0.043
Egg breaking rate, %	0.87	0.36	0.185	0.07
Egg shape index	1.32	1.32	0.007	0.71
Shell breaking strength, kg/cm^2^	3.76	4.01	0.128	0.21
Shell thickness, mm	0.36	0.36	0.005	0.91

Abbreviations: BY, monobutyrin group; CG, control group.

Butyrate in the gastrointestinal tract is able to improve growth performance by changing the nutrient digestibility, microbiota composition, and immune responses (Moquet, 2018). While assessing the effects of butyrate additives, different responses could be attributed to inclusion level, diet composition, age, and health status (Cerisuelo et al., 2014). The increased ratio of intestinal villus height to crypt depth could be the reason behind the improved growth performance owing to the increased absorptive surface (Hu and Guo, 2007; Qaisrani, 2014). Supplementing effects of butyrate glycerides on broiler chicken growth performance are highly variable. Some researchers reported no effects on growth performance (Leeson et al., 2005; Panda et al., 2009), whereas improvement on growth performance was observed (Antongiovanni et al., 2007). Similar to our results, Bedford et al. (2017) did not observe any significant differences in overall ADG or feed conversion rate with addition level of monobutyrin in the diets from 500 ppm to 3,000 ppm. Hu and Guo (2007) suggested that 500 mg sodium butyrate/kg was the optimum level of supplementation for chickens because increased BW gain during the periods from 0 to 21 d was observed. Yin et al. (2016) observed that feed efficiency was increased by 10% with butyrate glyceride supplementation and abdominal fat deposition was also reduced in 3-week-old broilers. Nollet et al. (2002) found that supplementing sodium butyrate at 500 mg/kg had no effect on the average egg weight, but the lay efficiency and feed conversion were improved.

A few more studies observed that butyrate supplementation benefited the shell strength. Hanna (2019) did not observe any effects of butyrate (550 mg/kg) on average egg production, egg weight, egg mass, mortality, feed intake, egg components, or BW of laying hens. But, the author observed increased egg shell strength. Butyrate (addition level of 185 mg/kg) can enhance the egg shell strength in old hens and decrease the number of misshapen eggs (Sengor et al., 2007), which is similar to our study in which the egg breaking rate tended to decrease with monobutyrin supplementation.

### Blood Biochemical Indexes and Antioxidant Parameters

Supplementation of monobutyrin did not affect any of the blood biochemical indexes analyzed other than total protein (*P* = 0.047; Table 3). Regarding the antioxidant parameters, none were affected by monobutyrin supplementation (*P* > 0.05). The increased concentration of serum total protein might be caused by higher absorption efficiency with monobutyrin supplementation as it was reported that butyrate supplementation can increase the ratio of intestinal villus height to crypt depth (Qaisrani, 2014). Supplementation of butyrate glyceride can decrease serum triglyceride and total cholesterol concentrations (Yang et al., 2018). Broiler supplemented with mix of monobutyrin and tributyrin had higher calcium concentrations and lower serum cholesterol levels compared with control birds (Bedford et al., 2017). However, this was not observed in our study. Calcium can help reduce cholesterol levels (Kanyinji and Maeda, 2010). In our study, the treatment group had a numerically higher number of calcium concentration compared with the control group but not statistically significant. Thus, the cholesterol concentrations were not different either between the 2 groups. Limited researches have been conducted on the blood biochemical indexes and antioxidant parameter, thus we are not be able to make further comparisons.

**Table 3:** Effects of monobutyrin supplementation on blood biochemical indexes and antioxidant parameters of broiler breeders.

Item	CG	BY	SEM	*P* Value
Blood biochemical indexes				
Total protein, g/L	8.02	8.24	0.070	0.047
Total cholesterol, mmol/L	6.88	8.21	0.955	0.35
Albumin, g/L	21.46	25.09	2.837	0.38
Triglyceride, mmol/L	18.37	16.29	3.045	0.64
Alkaline phosphatase, U/L	23.56	11.86	4.052	0.08
Calcium, mmol/L	3.78	3.83	0.212	0.87
Antioxidant parameters				
MDA, nmol/mL	5.98	4.76	0.764	0.28
T-AOC, mgprot	5.47	3.49	1.284	0.30
SOD, U/mL	4.73	4.16	0.258	0.15
GSH-PX, U/mL	1,415.6	1,249.5	89.15	0.22

Abbreviations: BY, monobutyrin group; CG, control group; GSH-PX: glutathione peroxidase; MDA, malondialdehyde; T-AOC, total antioxidant capacity; SOD, superoxide dismutase.

Yin et al. (2016) observed that mixed butyrate glycerides decreased fat deposition, and this corresponded with changes in serum lipid profiles and lipid metabolism–related enzymes. Yang et al. (2018) reported butyrate glyceride supplementation increased serum concentrations of alanine, low-density and very-low-density lipoproteins, and lipids. The study also found that butyrate supplementation boosted serum concentration of bacterial metabolite, including choline, dimethylamine, lactate, and succinate. The author indicated that potential contribution of intestinal bacteria to lipid metabolism/energy homeostasis through their metabolites in broilers existed. Bedford et al. (2017) reported that supplementation of monobutyrin and tributyrin affected the serum parameters related to muscle growth and fat deposition indicating that butyrate glycerides shifted lipid metabolism. Bedford et al. (2016) incorporated tributyrin into the broiler chicken diets and did not observe any effects on overall daily gain and feed conversion ratio. However, the hepatic gene expression and abdominal fat deposition were affected. The butyrate activity as a histone deacetylase inhibitor could be the reason to increase muscle fiber cross-sectional area and decrease intramuscular fat deposition (Walsh et al., 2015).

### Operational Taxonomic Unit Diversity, Similarity Analysis, and Alpha Diversity

**Figure 1:**
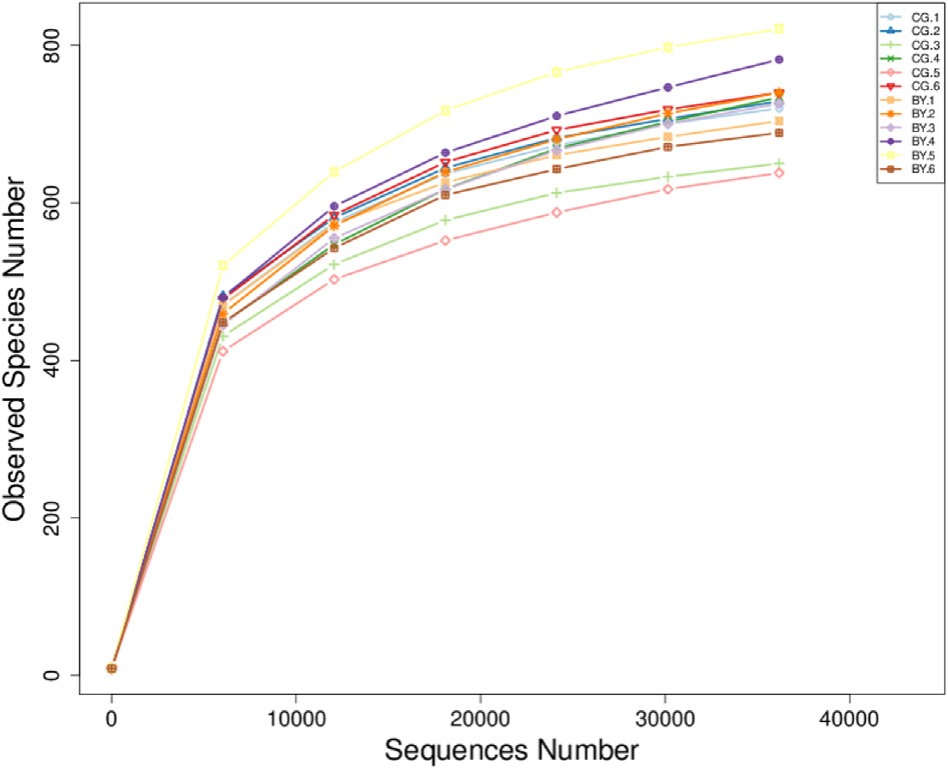
Rarefaction curves of number of operational taxonomic units (OTUs) in each group. Abbreviations: BY, monobutyrin group; CG, control group.

**Figure 2:**
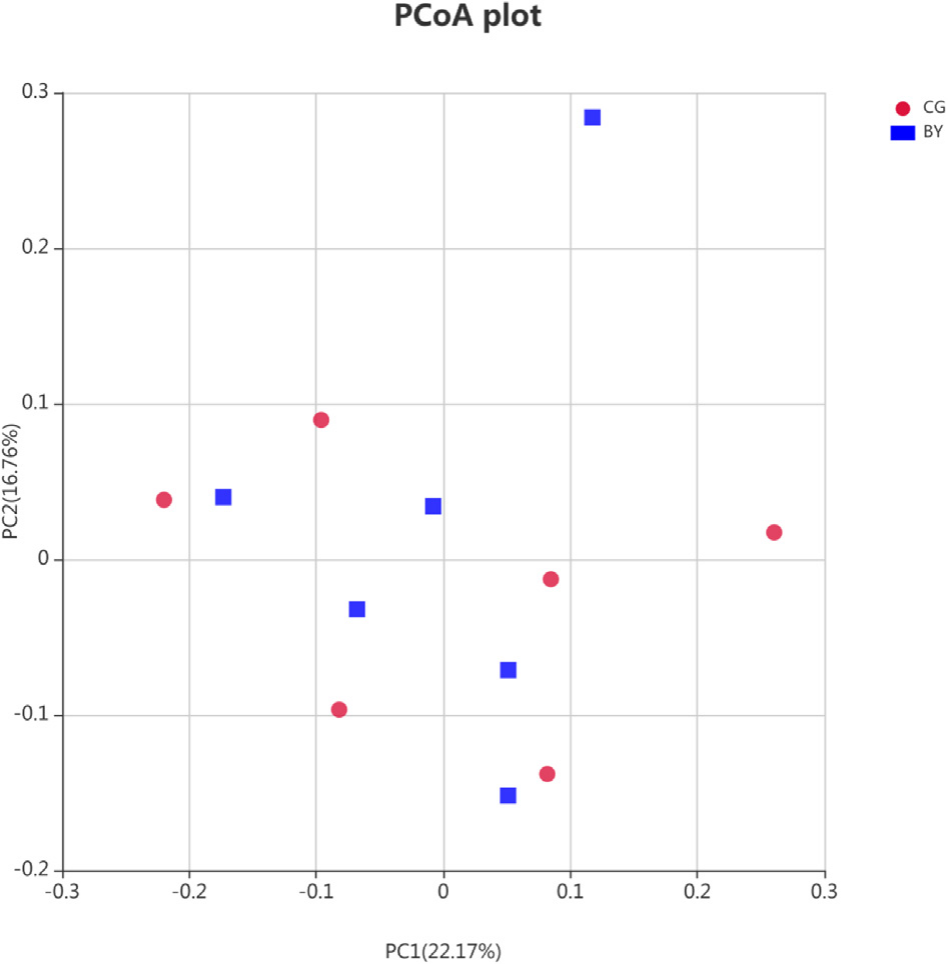
Principle coordinate analysis (PCoA) of the cecal microbiota in different groups. Abbreviations: BY, monobutyrin group; CG, control group; PC1, first principal component; PC2, second principal component.

After data filtering, quality control, and removal of chimera sequences, an average of 53,580 effective sequences were obtained for each sample. The length of the sequences ranged between 414 and 419 bp with an average length of 416 nucleotides. Rarefaction curve revealed that there was sufficient OTU coverage to describe the bacterial composition of each group (Figure 1). The overall number of OTU was 1301 and 974 shared OTU were detected in both groups. The sequence depth was sufficient enough to capture the majority of OTU in the cecal samples. Principal coordinate analysis using the Bray–Curtis similarity method revealed that the first principal component and the second principal component explained 22.17 and 16.76% of the variation in microbial diversity, respectively. As shown in Figure 2, no distinguishable clustering of samples appeared to be evident between the control and treatment groups (Figure 2). Few studies have been conducted on alpha diversity regarding butyrate glyceride supplementation. In our study, alpha diversity indexes including Shannon, Simpson, Chao1, Ace, and Good’s Coverage were not affected by monobutyrin supplementation (Table 4). Moquet (2018) reported that phylogenetic diversity (an alpha diversity index) and microbiota composition at the phylum level were affected by dietary supplementation of unprotected butyrate salt. Yang et al. (2018) pointed out that supplementing 3,000 ppm of butyrate altered intestinal microbiota composition, but it did not affect the alpha diversity, which was similar to our results.

**Table 4:** Effects of monobutyrin supplementation on on alpha diversity indexes of cecal microbiota of broiler breeders.

Item	CG	BY	SEM	*P* Value
Shannon	6.87	7.04	0.101	0.27
Simpson	0.97	0.98	0.003	0.28
Chao1	786.0	836.3	24.75	0.18
Ace	793.7	841.7	25.30	0.21
Goods_coverage	0.99	0.99	0.0002	0.38

Abbreviations: BY, monobutyrin group; CG, control group.

### Taxonomic Composition of Cecal Microbiota

**Figure 3:**
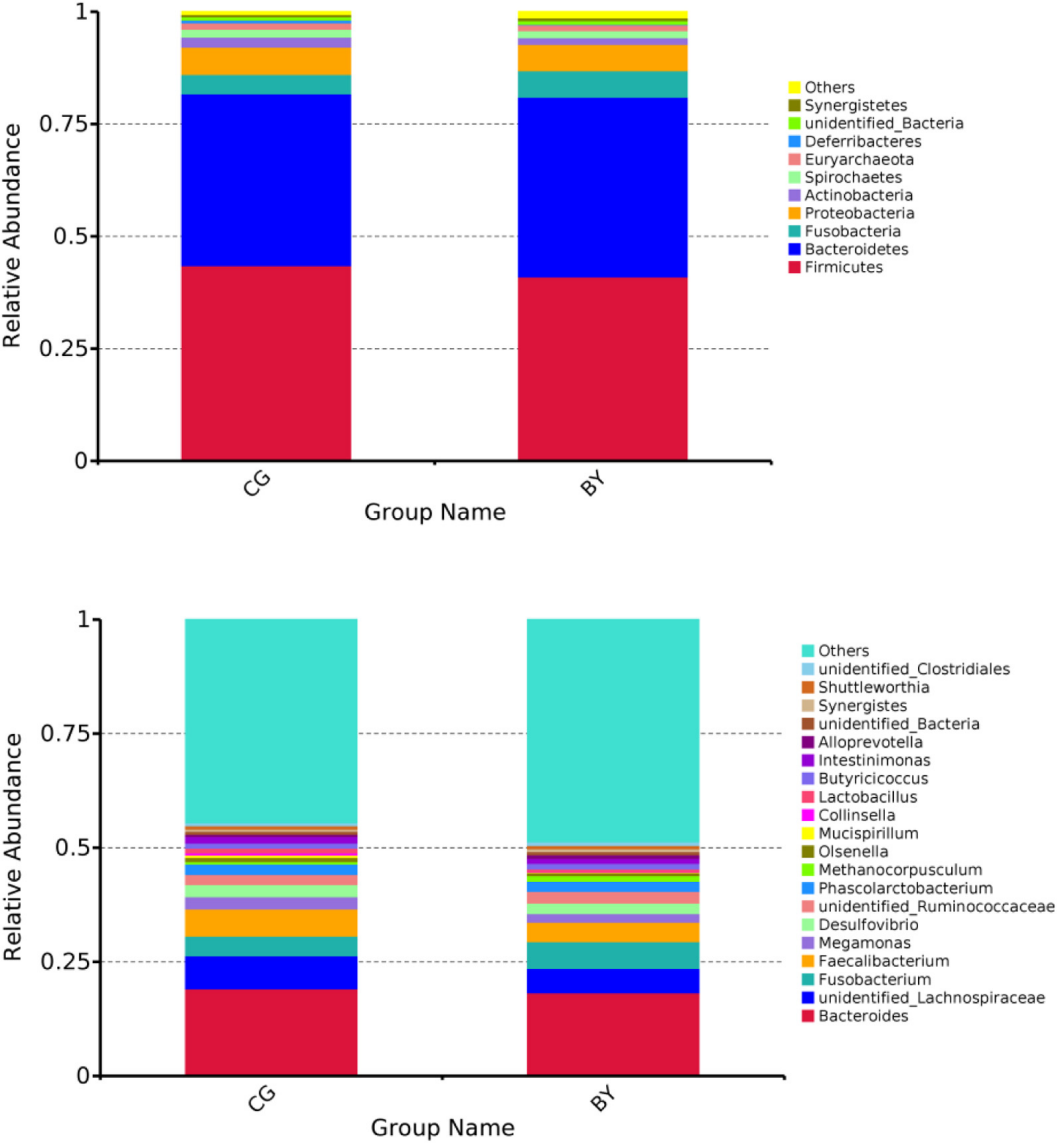
Phylum-level (top) and genus-level (bottom) taxonomic composition of the cecal microbiota in different groups. Abbreviations: BY, monobutyrin group; CG, control group.

The taxonomic composition of the cecal microbiota is presented in Table 5, Table 6, and Figure 3. At the phylum level, Firmicutes (>40%) and Bacteroidetes (>38%) are the first 2 most predominant phylum followed by Proteobacteria (>5%). At the genus level, *Bacteroides* was dominant (>18%), followed by *Lachnospiraceae* (>5%), *Fusobacterium* (4%), and *Faecalibacterium* (4%). The relative abundance of the rest genera listed is all lower than 4% (Table 6). Overall, the supplementation of monobutyrin did not affect microbiota composition at both phylum and genus levels (*P* > 0.05).

**Table 5:** Effects of monobutyrin supplementation on phylum level taxonomic compositon (%) of the cecal microbiota of broiler breeders.

Item	CG	BY	SEM	*P* Value
Firmicutes	43.54	41.03	3.004	0.57
Bacteroidetes	38.24	39.98	2.202	0.59
Proteobacteria	6.11	5.91	0.717	0.84
Fusobacteria	4.31	5.89	1.714	0.53
Actinobacteria	2.19	1.45	0.779	0.52
Spirochetes	1.75	1.54	0.801	0.86
Euryarchaeota	1.44	1.32	0.478	0.86
Deferribacteres	0.59	0.14	0.300	0.31
Synergistetes	0.48	0.62	0.189	0.61
Unidentified bacteria	0.77	0.76	0.223	0.97
Others	0.58	1.35	0.154	0.005

Abbreviations: BY, monobutyrin group; CG, control group.

**Table 6:** Effects of monobutyrin supplementation on genus level taxonomic compositon (%) of the cecal microbiota of broiler breeders.

Item	CG	BY	SEM	*P* Value
*Bacteroides*	19.11	18.22	2.132	0.77
Unidentified Lachnospiraceae	7.24	5.38	1.610	0.43
*Fusobacterium*	4.31	5.89	1.715	0.53
*Faecalibacterium*	5.95	4.22	1.300	0.36
*Megamonas*	2.72	1.88	0.693	0.41
*Desulfovibrio*	2.67	2.34	0.543	0.68
Unidentified Ruminococcaceae	2.18	2.54	0.289	0.39
*Phascolarctobacterium*	2.34	2.25	0.359	0.87
*Methanocorpusculum*	0.52	1.15	0.396	0.28
*Olsenella*	0.86	0.67	0.299	0.67
*Mucispirillum*	0.59	0.14	0.300	0.31
*Collinsella*	0.47	0.11	0.281	0.38
*Lactobacillus*	0.98	0.63	0.239	0.32
*Butyricicoccus*	1.15	1.23	0.185	0.76
*Intestinimonas*	1.41	1.07	0.131	0.10
*Alloprevotella*	0.46	0.77	0.169	0.22
Unidentified bacteria	0.77	0.76	0.223	0.97
Synergistes	0.48	0.62	0.189	0.61
*Shuttleworthia*	0.74	0.75	0.161	0.96
Unidentified Clostridiales	0.62	0.81	0.156	0.41
Others	44.52	48.61	2.069	0.19

Abbreviations: BY, monobutyrin group; CG, control group.

The dissociation of short-chain fatty acid in a bacterial cytoplasm can disrupt the proton motive force across the membrane and lower the cytoplasmic pH. This is one of the possible reasons that butyrate has bacteriostatic or bactericidal effects (Moquet, 2018). Previous studies indicated that butyrate supplementation benefit health and growth performance when the gut microbiota is disturbed (Bortoluzzi et al., 2017). Most studies regarding butyrate have been focusing on reducing specific pathogenic bacteria such as *Salmonella*. van Der Wielen et al. (2000) reported a decrease in coliform count and an increase in *Lactobacillus* count. Qaisrani (2014) reported reduced Shannon's diversity index as well as reduced relative abundance of *Clostridium perfringens* with supplementation of 2 g/kg fat-coated butyrate. Using *S.* Enteritidis–challenged birds as experimental animals, sodium butyrate prevented growth reduction in the treatment group birds compared with the control birds (Zhang et al., 2011). Panda et al. (2009) reported that supplementation of 4 and 6 g/kg unprotected butyrate in diet can reduce *Escherichia coli* in the crop and small intestine of broilers. However, Czerwinski et al. (2012) did not observe any effects on the total number of bacteria and *Lactobacillus* spp. as well as *Enterococcus* spp. counts in ileal and caecal digesta with fat-coated butyrate. The inconsistent results may be because of the inclusion level and forms of butyrate, diet composition, age, breed, and health status, as well as release locations of butyrate compounds.

Some bacteria in the cecum are related to feed conversion efficiency in broiler chickens such as *Lactobacillus* spp., Ruminococcaceae, Clostridiales, Gammaproteobacteria, Bacteroidales (ValeriaTorok and Ophel-Keller, 2011). The abundance of cecal *Lactobacillaceae* was significantly decreased with butyrate supplementation both in broilers and weaned piglets (Huang et al., 2015; Onrust et al., 2020). Hu and Guo (2007) observed that dietary supplementation of sodium butyrate decreased the *Lactobacillus* count linearly with increasing levels of supplementation. In our study, the relative abundance of *Lactobacillus* was not affected by monobutyrin supplementation (*P* = 0.32), although the treatment group had a lower number compared with the control group (0.63% vs. 0.98%). The inclusion level, basal diet, as well as health status all could be playing a role to cause these inconsistent results.

## Conclusions

Supplementation of monobutyrin increased egg weight and tended to decrease egg breaking rate of Qingyuan partridge chickens at the late stage of production. Alpha diversity indexes including Shannon, Simpson, Chao1, Ace, Good’s Coverage and composition of cecal microbiota were not affected by monobutyrin supplementation.
